# Targeting Ferroptosis, Pyroptosis, and NRF2 Signaling with Dietary Polyphenols in Diabetic Microvascular Complications: An Integrative Review

**DOI:** 10.3390/nu18172870

**Published:** 2026-09-02

**Authors:** Ana María García-Muñoz, Desirée Victoria-Montesinos, Juana M. Morillas-Ruiz

**Affiliations:** Faculty of Pharmacy and Nutrition, UCAM Universidad Católica de Murcia, Campus de los Jerónimos 135, Guadalupe, 30107 Murcia, Spain; amgarcia13@ucam.edu (A.M.G.-M.); jmmorillas@ucam.edu (J.M.M.-R.)

**Keywords:** diabetic retinopathy, diabetic kidney disease, diabetic peripheral neuropathy, ferroptosis, pyroptosis, NRF2, NLRP3 inflammasome, dietary polyphenols, oxidative stress, nutraceuticals

## Abstract

Diabetic retinopathy, diabetic kidney disease, and diabetic peripheral neuropathy remain major causes of visual loss, kidney failure, pain, disability, and reduced quality of life despite improvements in glycemic and cardiovascular risk management. Oxidative stress, mitochondrial dysfunction, iron dyshomeostasis, lipid peroxidation, and sterile inflammation are shared features of these complications and converge on regulated cell-death programs. Ferroptosis is driven by iron-dependent phospholipid peroxidation when glutathione peroxidase 4 and complementary antioxidant systems are insufficient, whereas pyroptosis is an inflammatory lytic process executed by gasdermins after activation of inflammasome-associated or other inflammatory caspases. Nuclear factor erythroid 2-related factor 2 (NRF2) connects these pathways by regulating glutathione synthesis, lipid peroxide detoxification, iron handling, mitochondrial homeostasis, and redox-sensitive inflammatory signaling. Dietary polyphenols may influence this network through electrophilic or kinase-dependent NRF2 activation, preservation of the SLC7A11-glutathione-GPX4 axis, modulation of iron and lipid metabolism, and inhibition of NF-kappaB, TXNIP, NLRP3, caspase-1, and gasdermin signaling. This integrative review critically examines mechanistic, preclinical, and human evidence for these effects in the diabetic retina, kidney, and peripheral nerve. The strongest direct preclinical evidence currently concerns corilagin, resveratrol, isoquercetin, quercetin, epigallocatechin gallate, punicalagin, and selected anthocyanin-rich or phenolic extracts. Human studies suggest possible benefits for albuminuria, retinal edema, endothelial function, and neuropathic outcomes, but they rarely measure ferroptosis- or pyroptosis-specific biomarkers and their results are heterogeneous. The proposed ferroptosis–pyroptosis–NRF2 network should therefore be viewed as a biologically plausible integrative framework rather than a clinically validated linear pathway. Future trials require chemically characterized interventions, exposure biomarkers, tissue-relevant pharmacokinetics, prespecified regulated-cell-death panels, and clinically meaningful microvascular endpoints.

## 1. Introduction

Diabetes mellitus is a growing global health challenge. The 11th edition of the International Diabetes Federation Atlas estimated that 589 million adults aged 20–79 years were living with diabetes in 2024 and projected that this number could rise to 853 million by 2050 [1]. The clinical burden extends far beyond chronic hyperglycemia because prolonged exposure to glucose variability, dyslipidemia, advanced glycation end products (AGEs), altered insulin signaling, and hemodynamic stress produces persistent biochemical injury in vascularized tissues [2,3,4]. Diabetic retinopathy (DR), diabetic kidney disease (DKD), and diabetic peripheral neuropathy (DPN) are classically categorized as microvascular complications, although each involves coordinated injury to vascular, neural, epithelial, stromal, and immune compartments [5,6,7].

Nevertheless, intensive control of glucose, blood pressure, and lipids reduces microvascular risk, but target-organ damage can continue after conventional risk factors improve. This residual risk is partly explained by metabolic memory, in which mitochondrial overproduction of reactive oxygen species (ROS), epigenetic remodeling, AGE-receptor signaling, protein kinase C activation, and impaired endogenous antioxidant responses perpetuate injury [2,3,4]. In the retina, oxidative stress interacts with neuroinflammation, endothelial activation, pericyte loss, and blood–retinal barrier failure [5,8]. In the kidney, podocyte depletion, endothelial dysfunction, mesangial expansion, tubular stress, and fibrosis jointly determine progression [4,6]. In peripheral nerves, metabolic injury is compounded by microvascular insufficiency, mitochondrial dysfunction, altered axonal transport, Schwann-cell dysfunction, and neuroimmune activation [7,9].

Against this background, these processes were historically described mainly in terms of apoptosis or nonspecific oxidative damage. The current concept of regulated cell death provides a more discriminating framework because it identifies molecularly controlled processes that can be measured and potentially modified. Ferroptosis is characterized by iron-dependent accumulation of peroxidized phospholipids, whereas pyroptosis is characterized by gasdermin-mediated membrane permeabilization and inflammatory mediator release [10,11,12]. Both are relevant to diabetes: hyperglycemia can impair glutathione-dependent lipid peroxide detoxification, disturb iron and mitochondrial homeostasis, prime inflammasomes, and increase the danger signals that activate inflammatory caspases [13,14,15].

Within this mechanistic network, NRF2 occupies a central position. Under stress, disruption of Kelch-like ECH-associated protein 1 (KEAP1)-dependent NRF2 degradation permits NRF2 accumulation and transcription of genes involved in glutathione synthesis, xenobiotic metabolism, NADPH regeneration, iron sequestration, and cellular repair [16,17,18]. NRF2 can therefore reduce susceptibility to ferroptosis, while its effects on mitochondrial ROS, thioredoxin-interacting protein (TXNIP), nuclear factor kappa B (NF-kappaB), and NLRP3 signaling may also restrain inflammasome activation and pyroptotic injury [19,20,21]. These actions are context-dependent, however, because excessive or prolonged heme oxygenase 1 (HO-1) activity can increase the labile iron pool and potentially favor ferroptosis when downstream iron-buffering capacity is exceeded [19,22].

Polyphenols were selected as the nutritional focus because they are common constituents of plant foods, but they are also administered as standardized extracts, purified compounds, and formulation-enhanced products. This spectrum makes it possible to examine the gap between habitual intake and pharmacological testing, while their convergent effects on redox adaptation, mitochondrial quality control, lipid peroxidation, and inflammasome signaling make them especially relevant to the interconnected pathways considered here.

Within this convergent framework, dietary polyphenols are particularly attractive because their biological activity is not limited to direct radical scavenging. Flavonoids, stilbenes, phenolic acids, tannins, lignans, and related phenolic compounds can affect NRF2, NF-kappaB, AMP-activated protein kinase (AMPK), sirtuins, mitogen-activated protein kinases, and the NLRP3 inflammasome [23,24,25]. Resveratrol, curcumin, quercetin, epigallocatechin gallate (EGCG), anthocyanins, corilagin, and punicalagin have each been associated with antioxidant, anti-inflammatory, endothelial-protective, or anti-fibrotic effects in diabetic complications [26,27,28]. Several of these compounds now have direct preclinical links to ferroptosis or pyroptosis, but the evidence is scattered across organs and experimental systems.

Accordingly, the aim of this integrative review is to critically synthesize evidence that dietary polyphenols can modulate ferroptosis, pyroptosis, and NRF2 signaling in DR, DKD, and DPN. Particular emphasis is placed on distinguishing direct evidence of regulated cell death from studies that measured only oxidative stress or inflammasome components, separating preclinical findings from human outcomes, and identifying the pharmacokinetic and methodological barriers that currently limit translation. The central premise is not that the three pathways form a single obligatory cascade, but that they represent an interacting redox-inflammatory network that may reveal tractable nutritional targets.

## 2. Review Scope and Literature Identification

### 2.1. Review Design and Search Approach

Accordingly, this work was designed as a narrative integrative review. Targeted searches were conducted in PubMed/MEDLINE from database inception to 3 August 2026. Search concepts combined the three canonical microvascular complications with terms related to ferroptosis, pyroptosis, inflammasomes, NRF2, and dietary polyphenols. Compound-specific searches included resveratrol, curcumin, quercetin, isoquercetin, EGCG, anthocyanins, punicalagin, corilagin, naringenin, vitexin, honokiol, carnosic acid, and phenolic-rich extracts. Reference lists of mechanistic reviews and key primary articles were screened to identify foundational studies and additional human trials. The main search framework is summarized in Table 1.

The named compounds were not defined a priori as an exhaustive panel. They were identified iteratively through the broader searches and retained when they met at least one of the following criteria: direct ferroptosis or pyroptosis evidence in a diabetic microvascular model; NRF2-linked mechanistic evidence in a relevant organ; or human data for a diabetic microvascular endpoint. Other phenolic candidates were included when they clarified pathway specificity or broadened the chemical classes represented.

### 2.2. Evidence Selection and Interpretation

Accordingly, peer-reviewed English-language studies were prioritized when they addressed at least one of the following: (i) molecular machinery or validated readouts of ferroptosis; (ii) inflammasome activation accompanied by gasdermin cleavage or other evidence of pyroptotic membrane execution; (iii) NRF2 pathway engagement; (iv) a dietary polyphenol or phenolic-rich intervention in a relevant diabetic tissue; or (v) a clinical microvascular endpoint. Human studies were retained even when they did not measure regulated-cell-death biomarkers because they inform translational plausibility. Non-polyphenolic agents were included only when they clarified causality, validated a pathway, or provided a useful comparator.

Within this framework, the synthesis is qualitative. No PRISMA flow diagram, formal risk-of-bias tool, or certainty-of-evidence grading was applied, and the review should not be interpreted as exhaustive. Mechanistic conclusions were weighted according to evidence level. Changes in ROS, malondialdehyde (MDA), inflammatory cytokines, or NLRP3 alone were not considered proof of ferroptosis or pyroptosis. Stronger ferroptosis evidence required a coherent marker pattern plus rescue by a ferroptosis inhibitor, genetic manipulation, or lipid-peroxidation-specific intervention. Stronger pyroptosis evidence required gasdermin cleavage or membrane-pore execution in addition to inflammasome and cytokine measurements.

## 3. Ferroptosis in the Diabetic Microvascular Environment

### 3.1. Core Machinery and Antioxidant Defense Systems

To begin, ferroptosis was initially defined as an iron-dependent, non-apoptotic form of cell death induced by inhibition of the cystine-glutamate antiporter system x_c^- [10]. Its proximate biochemical driver is uncontrolled peroxidation of polyunsaturated fatty acyl chains within membrane phospholipids. Acyl-CoA synthetase long-chain family member 4 (ACSL4) promotes incorporation of oxidation-prone fatty acids into phospholipids and is therefore a major determinant of ferroptosis sensitivity [29]. Lipoxygenases, cytochrome P450 oxidoreductase, mitochondrial ROS, and non-enzymatic iron-catalyzed reactions can then propagate lipid radicals until membrane integrity and ion homeostasis fail [12,13].

At the level of intracellular protection, the canonical defense is the SLC7A11-glutathione-GPX4 axis. SLC7A11 imports cystine in exchange for glutamate, intracellular cystine is reduced to cysteine, and cysteine supports glutathione synthesis. GPX4 uses reduced glutathione to convert phospholipid hydroperoxides into non-toxic lipid alcohols [12,30]. Suppression of SLC7A11 by p53, depletion of glutathione, inhibition or degradation of GPX4, and insufficient NADPH regeneration can each lower the threshold for ferroptosis [19,31]. In diabetes, this pathway is particularly vulnerable because chronic oxidative demand consumes glutathione while glycoxidative stress can impair antioxidant enzyme activity.

Importantly, ferroptosis is not controlled by GPX4 alone. Ferroptosis suppressor protein 1 (FSP1) regenerates reduced coenzyme Q at the plasma membrane and acts in parallel with GPX4 [32,33]. Dihydroorotate dehydrogenase (DHODH) provides a mitochondrial coenzyme Q-dependent defense, whereas the GTP cyclohydrolase 1-tetrahydrobiopterin pathway remodels lipids and supplies radical-trapping antioxidant capacity [34,35]. These complementary systems are relevant to polyphenol research because an intervention that restores GPX4 may still fail if iron loading or mitochondrial lipid oxidation remains uncontrolled, while apparent GPX4-independent protection may be mediated by FSP1, coenzyme Q, or tetrahydrobiopterin.

Alongside antioxidant defense, iron availability is the second indispensable component. Transferrin uptake, ferritin storage, ferroportin-mediated export, heme turnover, and ferritinophagy determine the size of the redox-active ferrous iron pool. Nuclear receptor coactivator 4-mediated ferritinophagy can release stored iron and increase ferroptosis susceptibility [36]. Diabetic tissues may accumulate iron through altered systemic handling, local hemorrhage, reduced export, heme degradation, or damaged mitochondria. Conversely, simple elevation of total tissue iron does not establish ferroptosis; it must be interpreted with lipid peroxide formation and failure of specific defense systems.

From a diagnostic perspective, ferroptotic cells often show smaller mitochondria, increased membrane density, and reduced cristae, but these features are not sufficiently specific for diagnosis. A rigorous experimental panel should combine labile iron, oxidized phospholipid or C11-BODIPY measurements, 4-hydroxynonenal, GPX4 activity or abundance, SLC7A11, ACSL4, and rescue by ferrostatin-1, liproxstatin-1, iron chelation, or genetic intervention [12,14]. Prostaglandin-endoperoxide synthase 2 transcripts, MDA, or general ROS should be treated as supportive rather than definitive markers.

### 3.2. How Diabetes Creates a Ferroptosis-Permissive State

Accordingly, persistent hyperglycemia increases mitochondrial electron leak, NADPH oxidase activity, AGE-RAGE signaling, and flux through the polyol and hexosamine pathways [2,3]. These processes reduce NADPH and glutathione availability, generate carbonyl and lipid-derived electrophiles, and damage respiratory-chain components. Insulin resistance and dyslipidemia can further enrich membranes with oxidation-prone lipids, while inflammation induces nitric oxide and ROS that amplify lipid radical propagation. The result is not necessarily immediate cell death; rather, diabetes lowers the ferroptosis threshold so that additional tissue-specific insults become decisive.

In addition to these redox pressures, mitochondrial dysfunction can contribute through ROS generation, impaired ATP production, altered tricarboxylic acid flux, and defective mitophagy. This is prominent in retinal cells, renal tubular cells, podocytes, neurons, and Schwann cells, all of which have high energetic or membrane-maintenance demands [37,38,39]. Endoplasmic-reticulum stress and TGF-beta signaling can also suppress SLC7A11 and couple fibrotic remodeling to ferroptotic vulnerability. In the kidney, this link is supported by reduced xCT and GPX4 in human diabetic biopsy material and by rescue of diabetic tubular injury with ferrostatin-1 [40].

As noted above, NRF2 normally counterbalances this environment by inducing genes required for glutathione synthesis, NADPH generation, quinone detoxification, ferritin formation, and iron export [16,19]. Diabetes may produce a biphasic response in which early NRF2 activation is compensatory, but chronic metabolic stress, epigenetic change, or disrupted nuclear signaling eventually renders the response insufficient. Because NRF2 is not uniformly suppressed in every cell or disease stage, absolute NRF2 abundance should be interpreted together with nuclear localization and transcriptional activity.

## 4. Pyroptosis and Inflammasome Signaling in Diabetes

### 4.1. Canonical, Non-Canonical, and Gasdermin-Dependent Pathways

In parallel, pyroptosis is an inflammatory form of lytic regulated cell death executed by gasdermin pores. In the canonical pathway, a sensor such as NLRP3 recruits the adaptor apoptosis-associated speck-like protein containing a caspase recruitment domain and pro-caspase-1. Active caspase-1 processes pro-interleukin (IL)-1 beta and pro-IL-18 and cleaves gasdermin D (GSDMD), releasing an *N*-terminal fragment that oligomerizes in membranes [11,41]. Pore formation drives ionic imbalance, cell swelling, release of inflammatory mediators, and eventual membrane rupture.

Beyond the canonical pathway, human caspase-4 and caspase-5, or murine caspase-11, directly sense cytosolic lipopolysaccharide in the non-canonical pathway and cleave GSDMD [42]. Potassium efflux generated by GSDMD pores can secondarily activate NLRP3, thereby linking non-canonical sensing to caspase-1 and cytokine maturation. Gasdermin E (GSDME) provides another route because caspase-3 cleavage can convert an apoptotic signal into a pyroptosis-like lytic phenotype [43]. Gasdermin pores can also permeabilize mitochondria and amplify caspase activation, illustrating that death programs are not always isolated modules [44].

Accordingly, the gasdermin family should be treated as the execution layer, whereas inflammasomes are upstream signaling platforms. NLRP3 activation without GSDMD cleavage may drive IL-1 beta secretion from living cells but does not prove pyroptosis. Conversely, GSDME-mediated lysis can occur without the canonical NLRP3-caspase-1 route. This distinction is particularly important in diabetes studies that infer pyroptosis from NLRP3, caspase-1, or cytokine expression alone.

### 4.2. Metabolic Activation of NLRP3

At the upstream signaling level, NLRP3 requires a priming signal that increases the transcription of NLRP3 and pro-IL-1 beta, commonly through Toll-like receptor or AGE-RAGE activation of NF-kappaB. A second activation signal then promotes inflammasome assembly. Relevant diabetic triggers include mitochondrial ROS, oxidized mitochondrial DNA, ATP-P2X7 receptor signaling, potassium efflux, lysosomal destabilization, ceramides, cholesterol crystals, and endoplasmic-reticulum stress [45,46,47].

Within this two-signal framework, TXNIP is a central redox-sensitive link. Under oxidative stress, TXNIP dissociates from thioredoxin and associates with NLRP3, facilitating inflammasome activation [20]. Hyperglycemia can increase TXNIP in retinal endothelial cells, podocytes, tubular cells, Schwann cells, and macrophages. Damaged mitochondria intensify this response by producing ROS and releasing mitochondrial danger signals, whereas effective mitophagy generally restrains it.

Consequently, pyroptosis is especially damaging in microvascular organs because it combines loss of specialized cells with local cytokine amplification. Endothelial or pericyte pyroptosis can increase barrier permeability, podocyte or tubular-cell pyroptosis can promote albuminuria and fibrosis, and Schwann-cell or macrophage pyroptosis can intensify neuroinflammation and nociceptive signaling [15,21,48]. IL-1 beta and IL-18 also recruit leukocytes, activate neighboring cells, and perpetuate a tissue environment that can further increase ROS, iron dysregulation, and lipid peroxidation. The principal distinctions among ferroptosis, pyroptosis, and NRF2-associated protection are summarized in Table 2.

## 5. NRF2 as a Convergent but Context-Dependent Regulatory Node

### 5.1. KEAP1-NRF2 Signaling and Metabolic Defense

At this nexus, under basal conditions, KEAP1 functions as a substrate adaptor for a CUL3-containing ubiquitin ligase complex that continuously targets NRF2 for degradation. Oxidative or electrophilic modification of reactive KEAP1 cysteines alters this process, allowing newly synthesized NRF2 to accumulate, enter the nucleus, heterodimerize with small Maf proteins, and bind antioxidant response elements [16,17]. NRF2-regulated genes include glutamate-cysteine ligase subunits, glutathione reductase, NQO1, thioredoxin reductase, ferritin chains, ferroportin, and enzymes that support NADPH production.

On this basis, polyphenols can activate this system through several non-exclusive routes. Electrophilic metabolites may modify KEAP1 cysteines, while upstream AMPK, SIRT1, PI3K-AKT, protein kinase C, and mitogen-activated kinases can alter NRF2 stability or nuclear trafficking [24,28]. Some polyphenols also reduce KEAP1 expression, promote p62-dependent sequestration of KEAP1, or improve mitochondrial function sufficiently to restore a more effective adaptive response. Because many parent polyphenols are rapidly conjugated, these effects may be mediated by metabolites rather than by the native dietary compound.

### 5.2. NRF2 and Ferroptosis

Accordingly, NRF2 can oppose ferroptosis at multiple levels: cystine uptake and glutathione synthesis, GPX4 substrate supply, detoxification of lipid-derived electrophiles, NADPH regeneration, ferritin-mediated iron storage, and ferroportin-mediated export [14,19]. This breadth explains why NRF2 activation often restores SLC7A11 and GPX4 while simultaneously reducing lipid ROS and iron-dependent injury in diabetic models. The effect is not universal, however. NRF2-driven HO-1 degrades heme and releases iron; if ferritin and ferroportin responses are inadequate, HO-1 may paradoxically expand the labile iron pool. Therapeutic interpretation therefore requires measuring iron handling rather than assuming that increased HO-1 is invariably anti-ferroptotic.

In DKD, this context dependence may vary with disease stage. During early hyperfiltration, increased glomerular and tubular metabolic workloads can produce a compensatory NRF2 response, and strengthening a coordinated antioxidant and iron-buffering program may preserve viable cells [4,6]. In advanced fibrotic DKD, nephron loss, hypoxia, and disturbed iron handling may uncouple HO-1 induction from adequate ferritin storage and ferroportin export; under these conditions, isolated or sustained HO-1 activation could enlarge the labile iron pool and increase lipid-peroxidation risk [19,22]. This stage-dependent model remains to be tested directly, so NRF2 nuclear activity, HO-1, ferritin, ferroportin, and labile iron should be assessed together.

### 5.3. NRF2 and Pyroptosis

Similarly, NRF2 does not directly replace the gasdermin execution machinery, but it can lower the probability of inflammasome activation. Reduced mitochondrial ROS and improved mitophagy limit NLRP3 activation signals, while NRF2-dependent antioxidant systems can restrain TXNIP and NF-kappaB signaling [20,21]. NRF2 and NF-kappaB also compete for transcriptional coactivators and can reciprocally influence inflammatory gene expression. In diabetic tissues, activation of an NRF2-HO-1-NLRP3 regulatory pattern has repeatedly been associated with lower caspase-1 activity and IL-1 beta release, although GSDMD is not always measured [22,24].

### 5.4. Crosstalk Between Ferroptosis and Pyroptosis

Bringing both arms together, ferroptosis and pyroptosis can coexist in the same diabetic organ without occurring in the same cell or at the same time. Ferroptotic lipid products and damage-associated molecular patterns may activate macrophages or neighboring cells, while pyroptotic cytokines and ROS can increase lipid peroxidation and disturb iron homeostasis. Mitochondrial injury, TXNIP, NF-kappaB, and NRF2 provide plausible points of convergence [13,15,25]. Nevertheless, direct demonstrations in which selective blockade of one pathway prevents execution of the other in a diabetic microvascular model remain uncommon.

Accordingly, ferroptosis, pyroptosis, and NRF2 are best understood as components of an interacting network. NRF2 may reduce susceptibility to both death programs, but this does not imply that ferroptosis causes pyroptosis or vice versa in every context. This distinction is essential when evaluating polyphenols: simultaneous reductions in lipid peroxidation, NLRP3, and cytokines suggest multi-target activity, but causal pathway mapping requires genetic or pharmacological dissection.

**Figure 1:**
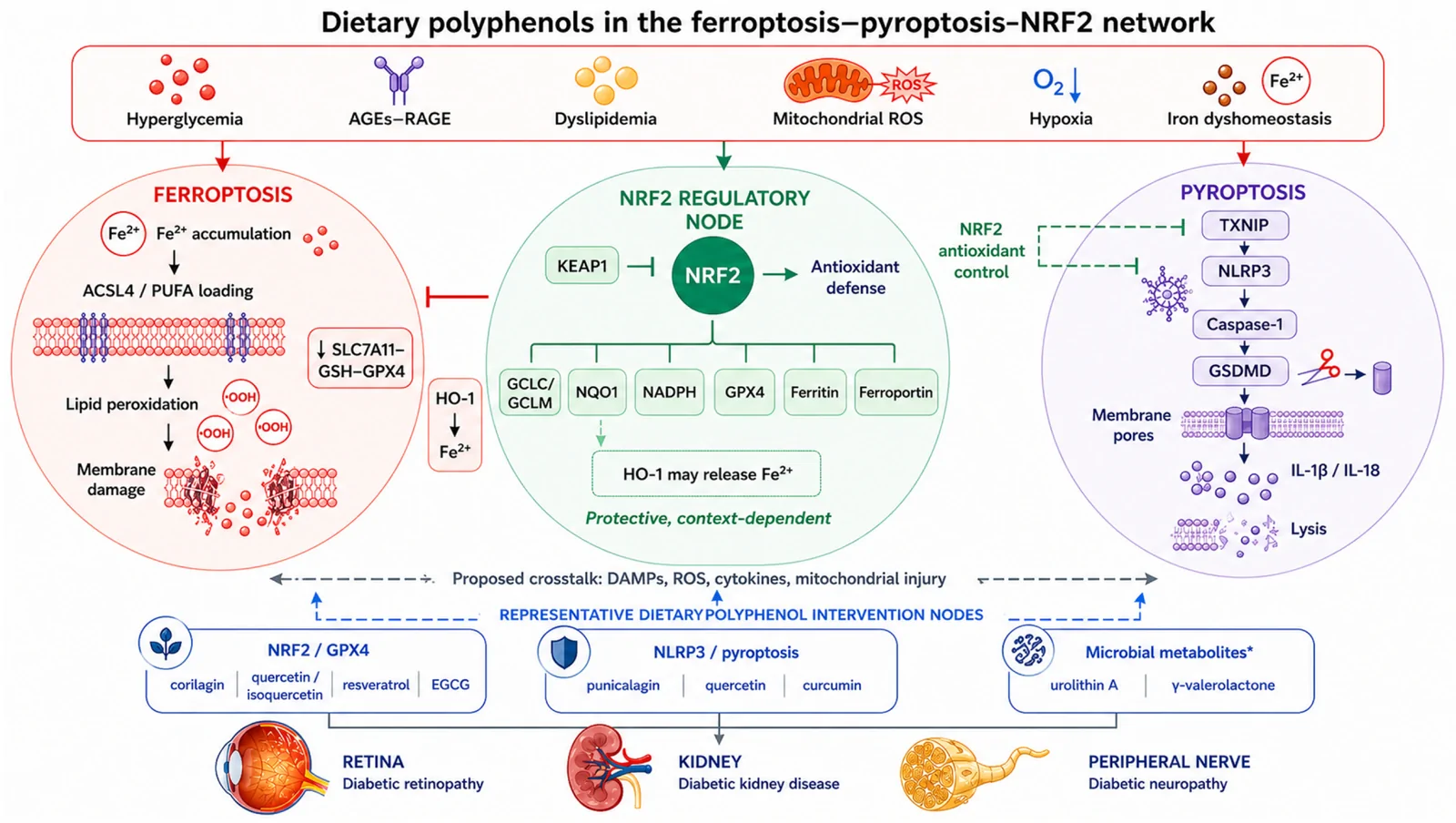
Conceptual network linking diabetic metabolic stress, ferroptosis, pyroptosis, NRF2, representative polyphenol intervention nodes, and the principal microvascular target tissues. Solid arrows denote established pathway relationships; dashed gray arrows denote proposed or indirect crosstalk; dashed blue arrows denote intervention links supported predominantly by preclinical evidence. NRF2 can support glutathione/GPX4 defenses and restrain redox-sensitive inflammasome signaling, whereas HO-1-associated iron release may become unfavorable if ferritin storage and ferroportin export are inadequate. Human studies have reported clinical or vascular signals but have not established pathway-mediated benefit. * Evidence concerning microbial metabolites is derived mainly from non-diabetic microvascular models.

Figure 1 provides an evidence-graded overview of these relationships, representative polyphenol intervention nodes, and the affected microvascular tissues.

## 6. Organ-Specific Evidence in Diabetic Microvascular Complications

### 6.1. Diabetic Retinopathy

At the organ level, the retina is exceptionally susceptible to redox injury because of its high oxygen consumption, abundant polyunsaturated lipids, continuous light exposure, and complex neurovascular organization. Hyperglycemia affects retinal endothelial cells, pericytes, Müller glia, microglia, retinal pigment epithelial cells, and neurons, making DR a neurovascular disease rather than an exclusively microvascular disease [5,8]. Barrier failure and microglial activation can expose different cell populations to iron, cytokines, and lipid radicals at different disease stages.

Consistent with this vulnerability, direct ferroptosis evidence has expanded rapidly. Ferrostatin-1 reduced retinal and cellular injury and improved the antioxidant capacity of the system ${X_{c}}^{-}$/GPX4 pathway in diabetic models [49]. Glial maturation factor-beta promoted early retinal ferroptosis by impairing chaperone-mediated autophagic degradation of ACSL4, linking glial stress to phospholipid remodeling [50]. Experimental data also indicate that ferroptosis contributes to retinal microvascular dysfunction, while mitochondrial GPX4 impairment and lipid peroxide accumulation may participate in retinal mitochondrial damage [37,51]. These findings support ferroptosis as more than a nonspecific oxidative endpoint, although clinical confirmation remains limited.

Beyond these general observations, barrier-specific mechanisms are also emerging. Flotillin-1 promoted NRF2 nuclear signaling and the SLC7A11-GPX4 defense, thereby reducing ferroptotic disruption of the blood–retinal barrier [52]. MicroRNA-214-3p attenuated damage through the p53-SLC7A11-GPX4 axis [53]. These studies underscore that regulation of ferroptosis differs by cell type and may be modified by non-coding RNAs, membrane scaffolds, autophagic flux, and local lipid composition.

In parallel, pyroptosis is supported by evidence of P2X7-NLRP3 activation in retinal endothelial cells and by gasdermin-associated inflammatory injury across the retinal neurovascular unit [47,54]. Extracellular ATP released during hyperglycemic stress can activate P2X7 receptors, promote potassium efflux, and amplify NLRP3 signaling. Müller glia and microglia can sustain cytokine production, while endothelial pyroptosis directly compromises vascular integrity. Studies that show NLRP3 or IL-1 beta changes without gasdermin cleavage remain relevant to inflammasome biology but should not be labeled as definitive pyroptosis.

Accordingly, several polyphenols have shown protective effects. Corilagin directly reduced retinal ferroptosis through NRF2 activation [55]. Resveratrol protected retinal cells through SIRT1-HMGB1-associated regulation of ferroptosis and has also been linked to NRF2-GPX4 signaling [56]. Isoquercetin inhibited p53-mediated ferroptosis, while quercetin reduced NLRP3-related inflammation and induced HO-1 in diabetic retinas [57,58,59]. Curcumin preserved NRF2 homeostasis and reduced oxidative retinal injury, although the cited study did not establish ferroptosis as the sole mode of cell death [60]. Carnosic acid, a phenolic diterpene, provided neurovascular protection through SIRT1-related signaling but likewise requires additional pathway-specific validation [61].

Beyond these individual compounds, anthocyanin-rich interventions act through complementary mechanisms. Blueberry anthocyanin extract reduced endoplasmic-reticulum stress through a microRNA-182-OGG1 axis, and cyanidin-3-O-glucoside reduced vascular leakage associated with microglial activation [62,63]. Naringenin reduced oxidative stress and apoptosis and improved neurotrophic signaling in diabetic rat retina [64]. These studies support retinal protection but do not by themselves demonstrate direct inhibition of ferroptosis or pyroptosis.

At the translational level, human evidence is suggestive but heterogeneous. Small controlled studies of French maritime pine bark procyanidins reported improvements in retinal circulation, edema, permeability, or visual acuity [65,66]. A combined pycnogenol–vitamin E–coenzyme Q10 supplement reduced circulating ROS and central macular thickness in non-proliferative DR, but the multicomponent design prevents attribution to polyphenols alone [67]. An open-label curcumin-phospholipid pilot study reported improvements in macular edema, whereas a six-month randomized trial of maltodextrinated grape pomace extract found improvements in retinal thickness, visual acuity, perfusion stability, and systemic oxidative markers [68,69]. None of these trials tested GPX4, ACSL4, cleaved GSDMD, or other pathway-specific endpoints.

### 6.2. Diabetic Kidney Disease

Beyond the retina, DKD is a multicompartment disorder involving the glomerular endothelium, podocytes, mesangial cells, tubular epithelium, pericytes, fibroblasts, and immune cells. Albuminuria reflects glomerular barrier dysfunction, but tubular injury and interstitial fibrosis are major determinants of long-term functional decline [4,6]. The kidney also handles filtered iron, heme proteins, and xenobiotic metabolites, which may increase local exposure to redox-active species.

Within this multicompartment context, ferroptosis has been demonstrated in diabetic tubular injury. Human diabetic kidney samples showed lower xCT and GPX4 transcripts, while diabetic mice and TGF-beta1-treated tubular cells showed glutathione depletion, lipid peroxidation, and protection by ferrostatin-1 [40]. An independent study similarly identified ferroptosis in renal tubular cell death under diabetic conditions [70]. Upregulation of NRF2 delayed DKD progression by inhibiting ferroptosis, supporting a causal protective role for this transcriptional response [71]. Podocyte ferroptosis is also biologically plausible because these cells have limited regenerative capacity and require sustained mitochondrial and cytoskeletal function.

Complementing these findings, pyroptosis contributes through both canonical and non-canonical routes. Caspase-11/4-GSDMD signaling promoted podocyte injury in experimental DKD [72]. Additional work has characterized GSDMD-mediated pyroptosis as a critical mechanism linking renal inflammation to structural injury [73]. Tubular and glomerular cells exposed to high glucose can activate TXNIP-NLRP3, while infiltrating macrophages provide further IL-1 beta and IL-18. Importantly, NLRP3 inhibition has not been uniformly beneficial in every interventional model, emphasizing that timing, cell specificity, and compensatory immune responses matter.

Within the anti-ferroptotic evidence base, quercetin provides some of the strongest direct evidence in DKD. It improved diabetic kidney injury through NRF2-HO-1 activation and reduced ferroptosis markers in tubular cells and diabetic rodents [74,75]. EGCG disrupted KEAP1-dependent NRF2 repression and reduced oxidative and fibrotic injury [76]. Vitexin and naringenin have also been linked to GPX4-related ferroptosis suppression or SIRT1-FOXO3a signaling [77,78]. Baicalin activated NRF2 and modulated MAPK signaling, although its study focused primarily on oxidative stress and inflammation rather than definitive ferroptotic rescue [79].

On the pyroptosis side, punicalagin provides some of the most direct evidence available. It inhibited TXNIP-NLRP3 signaling, reduced caspase-1/GSDMD-related responses, and protected against diabetic nephropathy in experimental models [80]. Neochlorogenic acid suppressed inflammation and pyroptosis, while honokiol reduced mitochondrial ROS-driven pyroptosis through SIRT3 [81,82]. Loganin, an iridoid rather than a polyphenol, reduced NLRP3-mediated pyroptosis and is useful as a mechanistic comparator [83]. Carnosine similarly reduced caspase-1-mediated podocyte pyroptosis but is not a polyphenol [84].

Beyond these more pathway-specific candidates, resveratrol engages several relevant pathways. It reduced oxidative stress and renal inflammatory cytokines through NRF2-KEAP1 signaling in diabetic animals [85]. Curcumin-piperine treatment has been associated with butyrate modulation and NRF2-HO-1 activation, suggesting a gut-kidney component that warrants metabolite-resolved investigation [86]. These multi-level effects may be advantageous, but they make it difficult to determine whether direct renal exposure, systemic metabolic improvement, or microbial metabolites are the primary drivers.

At the clinical level, the human DKD literature is more developed than that of other complications. Twelve weeks of green tea polyphenols containing 800 mg EGCG reduced residual albuminuria in participants receiving renin-angiotensin-system blockade [87]. Turmeric supplementation reduced proteinuria, TGF-beta, and IL-8 in a small randomized trial, while a short curcumin intervention reduced albuminuria and increased lymphocyte NRF2/NQO1 signaling [88,89]. Resveratrol at 500 mg/day for 90 days reduced albuminuria without significantly changing estimated glomerular filtration rate, and 400 mg/day improved brachial flow-mediated dilation in a crossover trial of patients with diabetes and stage 3 chronic kidney disease [90,91]. Meta-analytic evidence suggests modest improvements in albuminuria and selected oxidative or inflammatory biomarkers, but effects on filtration and hard kidney outcomes remain uncertain [92,93].

### 6.3. Diabetic Peripheral Neuropathy

A third setting, DPN, reflects injury to long axons, dorsal-root-ganglion neurons, Schwann cells, endoneurial microvessels, and neuroimmune networks. The high lipid content of myelin and the energetic demands of axonal transport create a plausible substrate for lipid peroxidation and ferroptosis [7,9]. At the same time, macrophage activation, Schwann-cell stress, and impaired mitophagy can activate inflammasomes and amplify pain or axonal degeneration [39,48].

Within this vulnerable cellular environment, direct evidence indicates that high glucose induces Schwann-cell ferroptosis by suppressing NRF2-related antioxidant defenses [94]. Honokiol reduced high-glucose-induced ferroptosis while activating AMPK-SIRT1-PGC-1alpha signaling in Schwann cells [95]. These observations suggest that mitochondrial biogenesis and ferroptosis defense may be coupled in DPN, but confirmation in human nerve tissue is lacking.

In parallel, pyroptosis evidence is centered on Schwann cells. Loganin reduces ROS generation, NLRP3 activation, and Schwann-cell pyroptosis under high-glucose conditions [96]. Reviews of DPN identify P2X7, NLRP3, caspase-1, GSDMD, IL-1 beta, and IL-18 as candidate targets across Schwann cells, macrophages, and neural tissue [48]. As in other organs, studies measuring only cytokines or NLRP3 cannot determine whether reduced neuroinflammation reflects true inhibition of pyroptotic execution.

Beyond the evidence tied directly to ferroptosis or pyroptosis, polyphenols have a broader preclinical history in neuropathy. Resveratrol improved functional, sensorimotor, and biochemical abnormalities in experimental DPN, and curcumin reduced spinal NADPH-oxidase-associated oxidative stress [97,98]. Quercetin, resveratrol, curcumin, and other dietary phenolics may influence nerve blood flow, aldose reductase, neurotrophic signaling, mitochondrial function, and inflammatory pathways [99]. The challenge is to determine which of these benefits depend on ferroptosis or pyroptosis rather than on general metabolic improvement.

At the clinical level, human findings are inconsistent and uninformative. Eight weeks of 80 mg/day nanocurcumin reduced neuropathy severity in one randomized trial and improved depression and anxiety scores in a related study population [100,101]. By contrast, a larger 16-week randomized trial using 40 mg twice daily did not improve pain, neurological examination scores, metabolic variables, or cardiovascular parameters [102]. A 12-week cocoa-polyphenol trial likewise did not demonstrate significant superiority over placebo for clinical or somatosensory outcomes [103]. These discordant results emphasize the need for adequately powered trials, standardized phenotyping, objective nerve-conduction or small-fiber outcomes, and exposure verification.

### 6.4. A Supporting Model: Diabetic Wound and Microvascular Repair

Similarly, diabetic wound healing is not one of the three canonical microvascular complications, but it integrates endothelial dysfunction, macrophage polarization, impaired angiogenesis, oxidative stress, neuropathy, and defective tissue repair. Experimental inhibition of ferroptosis improved diabetic wound healing through NRF2 activation [104]. This model supports the broader relevance of ferroptosis–NRF2 interactions in diabetic vascular repair, while cautioning against direct extrapolation to the retina, kidney, or nerve. The organ-specific evidence is summarized in Table 3.

## 7. Dietary Polyphenols as Multi-Target Modulators

### 7.1. From Radical Scavengers to Signaling Modulators

Within this network, polyphenols contain one or more phenolic rings and are commonly grouped as flavonoids, phenolic acids, stilbenes, lignans, and tannins. Their catechol, galloyl, or conjugated structures can donate electrons or hydrogen atoms and chelate transition metals in chemical systems. In vivo, however, direct scavenging is constrained by low tissue concentrations and extensive metabolism [105,106]. Signaling, enzyme modulation, membrane interactions, and the effects of microbial metabolites are therefore likely to be at least as important as stoichiometric radical neutralization.

Importantly, the label “dietary” describes chemical origin, not experimental dose equivalence. Many cell studies use micromolar parent-compound concentrations and animal studies use purified-compound doses that cannot be reproduced through habitual food intake. Throughout this review, habitual intake from foods or beverages, standardized extracts, purified compounds or conventional supplements, and formulation-enhanced products are therefore treated as distinct exposure categories [105,106].

Extending this signaling perspective, a recent integrative review highlighted the capacity of polyphenols to coordinate mitochondrial DNA maintenance, mitochondrial biogenesis, and mitophagy [107]. These mitochondrial-quality-control mechanisms are relevant here because damaged mitochondria can supply lipid-oxidation and inflammasome-activating signals, while NRF2, AMPK, sirtuins, and PGC-1alpha connect organelle renewal with cellular redox adaptation.

Accordingly, potentially relevant anti-ferroptotic actions include NRF2 activation, restoration of glutathione, maintenance of GPX4 and SLC7A11, inhibition of ACSL4-dependent lipid remodeling, chelation or redistribution of iron, preservation of mitochondria, and direct trapping of lipid radicals. For pyroptosis, polyphenols may reduce NF-kappaB-dependent priming, TXNIP-NLRP3 assembly, mitochondrial ROS, P2X7 signaling, caspase-1 activation, or gasdermin cleavage [24,25]. A compound should be described as anti-ferroptotic or anti-pyroptotic only when pathway-specific evidence supports that designation.

### 7.2. Resveratrol

Among the individual compounds reviewed, resveratrol is a stilbene found in grapes, berries, peanuts, and wine. Its signaling profile includes SIRT1, AMPK, PGC-1alpha, NRF2, and NF-kappaB, providing plausible links to mitochondrial quality control, glutathione metabolism, and inflammasome restraint. In DR models, resveratrol reduced ROS and inflammation through SIRT1-HMGB1-associated ferroptosis regulation [56]. In diabetic kidney models, it activated NRF2-KEAP1-related defenses and reduced inflammatory cytokines [85]. Experimental DPN studies also report functional and biochemical neuroprotection [97].

Despite this broad mechanistic profile, translation is limited by rapid absorption followed by extensive glucuronidation and sulfation, yielding very low circulating concentrations of unconjugated resveratrol [108]. This does not imply biological inactivity because conjugates and microbial metabolites may be deconjugated or act independently, but it does invalidate direct comparison with many micromolar in vitro experiments. Human renal studies show that 400–500 mg/day can affect albuminuria or endothelial function, yet neither outcome establishes direct ferroptosis or pyroptosis modulation [90,91].

### 7.3. Curcumin

Likewise, curcumin is the principal diarylheptanoid of turmeric. It can influence KEAP1-NRF2, NF-kappaB, NLRP3, mitochondrial function, and fibrotic signaling. In diabetic retina, curcumin preserved NRF2 pathway homeostasis and reduced oxidative injury [60]. In DKD, turmeric or curcumin interventions have reduced albuminuria and inflammatory mediators, and one human study demonstrated increased NRF2/NQO1 signaling in blood lymphocytes [88,89]. Curcumin also reduced oxidative injury in experimental neuropathy [98].

As with resveratrol, native curcumin has poor aqueous solubility, limited absorption, and rapid metabolism [109]. Nanoparticles, phospholipid complexes, piperine combinations, and other delivery systems can markedly change exposure, so findings cannot be generalized across formulations. Clinical DPN trials illustrate this problem: an 8-week nanocurcumin study was favorable, but a 16-week trial at a nominally similar daily dose was negative [100,102]. Formulation composition, baseline disease severity, adherence, and outcome selection may explain part of the difference.

### 7.4. Quercetin, Isoquercetin, and Related Flavonols

Within the flavonol class, quercetin is abundant in onions, apples, berries, capers, and tea. Its conjugates and glycosides differ substantially in terms of absorption and tissue delivery. In DKD models, quercetin activated NRF2-HO-1, restored ferroptosis defenses, and reduced renal injury [74,75]. In the diabetic retina, quercetin induced HO-1 and reduced NLRP3-associated inflammation, while isoquercetin directly inhibited p53-mediated ferroptosis [57,58,59]. These effects make the quercetin family a strong candidate for comparative pharmacokinetic and pathway studies.

Nevertheless, the HO-1 finding requires careful interpretation. An NRF2-HO-1 response may be protective when ferritin, ferroportin, and NADPH-dependent systems handle the released iron, but it could be unfavorable when iron sequestration is saturated. Future quercetin studies should therefore measure the labile iron pool and ferritinophagy alongside HO-1, GPX4, and lipid oxidation.

### 7.5. Catechins and Green Tea Polyphenols

Among catechins, EGCG is the dominant catechin in green tea and contains both catechol and gallate motifs. In experimental DKD, EGCG disrupted KEAP1 repression, increased NRF2 activity, and reduced oxidative and fibrotic injury [76]. A randomized clinical trial of a green tea polyphenol preparation containing 800 mg EGCG reduced residual albuminuria over 12 weeks [87]. The clinical result is relevant but does not clarify whether podocyte protection involved NRF2, WNT signaling, ferroptosis, or multiple pathways.

Despite this clinical signal, high-dose concentrated green tea extracts are pharmacologically different from habitual tea consumption. Exposure varies with fasting state, matrix, and formulation, and safety monitoring is especially important for people with kidney or liver disease. Trials should report the full catechin profile rather than only the nominal EGCG dose.

### 7.6. Anthocyanins and Proanthocyanidin-Rich Extracts

Beyond the single-compound evidence, anthocyanin-rich interventions from berries, grapes, and pigmented plant foods may support endothelial and retinal function through antioxidant signaling, microglial modulation, and barrier protection. Blueberry anthocyanins and cyanidin-3-O-glucoside reduced diabetic retinal injury through endoplasmic reticulum, DNA repair, vascular leakage, and microglial pathways [62,63]. French maritime pine bark extract and grape pomace formulations provide human signals for retinal edema and visual outcomes [65,69].

However, these preparations are chemically complex. Procyanidin chain length, anthocyanin glycosides, phenolic acids, and non-phenolic constituents may all influence activity. Benefits should therefore be attributed to the characterized extract rather than to a single presumed constituent. Direct measurements of ferroptosis and pyroptosis are largely absent from these clinical studies.

### 7.7. Hydrolyzable Tannins and Other Phenolic Candidates

Finally, within this broader chemical landscape, corilagin and punicalagin are hydrolyzable tannins with unusually direct pathway evidence. Corilagin activates NRF2 and reduces ferroptosis in DR, whereas punicalagin inhibits TXNIP-NLRP3-associated pyroptosis in DKD [55,80]. Vitexin, naringenin, baicalin, neochlorogenic acid, honokiol, and carnosic acid broaden the chemical space by engaging GPX4, SIRT1-FOXO3a, NRF2-MAPK, inflammasome, SIRT3, or SIRT1 pathways [77,81,82].

At the same time, not all natural products used as mechanistic comparators are polyphenols. Loganin is an iridoid glycoside and carnosine is a dipeptide, despite evidence that they can inhibit NLRP3- or caspase-1-associated pyroptosis [83,84]. Maintaining chemical classification is important for a nutrition-focused review and prevents the category of dietary polyphenols from becoming synonymous with all plant-derived or natural compounds. Representative dietary polyphenols and phenolic compounds are summarized in Table 4.

## 8. Translation to Human Nutrition and Clinical Practice

### 8.1. Bioavailability, Metabolism, and Tissue Exposure

Clinically, polyphenol exposure is determined by food matrix, dose, intestinal transport, phase II conjugation, enterohepatic cycling, renal clearance, and gut microbial metabolism [105,106,110]. Parent compounds that dominate in vitro research may be absent or present only transiently in plasma. Glucuronides, sulfates, methylated products, valerolactones, urolithins, and smaller phenolic acids can have different tissue distributions and biological effects.

Specific metabolite-level evidence remains preliminary and comes mainly from models outside of diabetic microvascular complications. Urolithin A, an ellagitannin-derived microbial metabolite, has inhibited TXNIP-NLRP3 signaling and pyroptosis in macrophage and inflammatory models and, in a separate acute-lung-injury model, increased SLC7A11 and GPX4 through NRF2-associated signaling. The proanthocyanidin metabolite 5-(3′,4′-dihydroxyphenyl)-γ-valerolactone, when combined with curcumin in primary microglia, reduced NLRP3 expression and modulated NOX2/NRF2 at low-micromolar concentrations [111,112,113]. These findings support metabolite-aware testing but cannot yet be extrapolated to diabetic retina, kidney, or peripheral nerve.

Crucially, tissue exposure is especially difficult to infer for microvascular organs. The blood–retinal barrier limits access to retinal tissue; CKD changes renal clearance and metabolite exposure; and peripheral nerves are protected by the blood–nerve barrier. Plasma concentration cannot be assumed to represent exposure in Müller cells, podocytes, tubular cells, or Schwann cells. Studies should measure parent compounds and major metabolites, ideally with tissue or target-engagement biomarkers.

In addition to host metabolism, the microbiota may contribute both metabolites and anti-inflammatory effects. Polyphenols can reshape microbial ecology, and microbial metabolism can generate compounds with greater systemic exposure than the parent molecule [110]. Curcumin-piperine-associated changes in butyrate and DKD provide an example of a possible gut-kidney mechanism [86]. Similar gut-retina and gut-nerve interactions are plausible but incompletely defined.

### 8.2. Human Evidence: Signals, Inconsistency, and Endpoint Limitations

These exposure constraints help explain why the clinical evidence summarized in Table 5 is hypothesis-supporting rather than definitive. In DKD, albuminuria has improved in trials of green tea polyphenols, turmeric/curcumin, and resveratrol [87,88,90]. Albuminuria is clinically relevant, but it can change without structural recovery or preservation of long-term filtration. Endothelial function improved with resveratrol in CKD and diabetes, but this systemic vascular endpoint is not equivalent to renal ferroptosis inhibition [91].

Likewise, in DR, several trials report improvements in edema, retinal thickness, perfusion, or visual acuity, but sample sizes are often small and formulations vary [65,68,69]. In DPN, the contrast between favorable short-term nanocurcumin findings and negative longer-term nanocurcumin and cocoa trials illustrates the uncertainty [100,102,103]. Publication bias, selective outcomes, short follow-up, baseline heterogeneity, and limited blinding in pilot studies may inflate the apparent benefit.

Despite these organ-specific signals, no clinical study establishes that a dietary polyphenol improves a microvascular outcome by simultaneously suppressing ferroptosis and pyroptosis through NRF2. Peripheral blood NRF2 levels or systemic MDA cannot substitute for tissue pathway evidence. The current clinical case is therefore indirect: the interventions can modify relevant oxidative, inflammatory, vascular, or clinical endpoints, while preclinical studies provide the regulated-cell-death mechanisms.

### 8.3. Safety and Integration with Standard Care

Regarding safety, polyphenol-rich foods can be incorporated within established healthy dietary patterns, but concentrated extracts and nanoformulations should be treated as bioactive interventions rather than as nutritionally interchangeable foods. Kidney impairment can alter metabolite clearance, while antiplatelet, anticoagulant, antihypertensive, and glucose-lowering therapies create the potential for interactions. Trials should prespecify adverse-event surveillance, liver and kidney chemistry, hypoglycemia, bleeding events, and concomitant medication changes.

Accordingly, polyphenols should not be presented as replacements for retinal screening, renin-angiotensin-system blockade, sodium-glucose cotransporter 2 inhibition, non-steroidal mineralocorticoid receptor antagonism, anti-vascular endothelial growth factor therapy, neuropathic pain management, or individualized glycemic control. The realistic near-term role is adjunctive and mechanism-informed.

### 8.4. Biomarkers and Trial Design

Accordingly, future trials need to link exposure, target engagement, and clinical response. A single systemic oxidative-stress marker is insufficient. A feasible panel could combine plasma or urinary oxylipins and 4-hydroxynonenal adducts with GPX4 activity, SLC7A11-related measures, ferritin/iron indices, cleaved GSDMD or gasdermin-derived extracellular-vesicle cargo, caspase-1 activity, IL-18, NRF2 nuclear activity in accessible cells, and metabolomic confirmation of polyphenol exposure. Tissue-specific fluids, such as urine and aqueous humor, may be more informative than plasma when ethically available.

Complementing these biomarker panels, clinical endpoints should match the organ and disease stage: optical coherence tomography, retinal leakage, and visual function for DR; UACR slope, measured or estimated filtration, tubular-injury markers, and kidney failure outcomes for DKD; and nerve conduction, intraepidermal nerve-fiber density, corneal confocal microscopy, quantitative sensory testing, and validated pain/function measures for DPN. Repeated measures are necessary because regulated-cell-death pathways may change before anatomical changes or symptoms. The proposed translational framework is summarized in Table 6.

## 9. Critical Gaps and Future Directions

The preceding evidence reveals several recurring limitations. First, direct evidence of simultaneous ferroptosis and pyroptosis modulation remains sparse. Many studies measure one complete pathway and only fragments of the other. Factorial experiments using selective inhibitors, CRISPR-based perturbations, cell-specific knockout models, and temporal sampling are needed to establish whether polyphenols act on parallel programs or on a causal sequence.

Second, cellular heterogeneity must be addressed. Single-cell transcriptomics, spatial proteomics, lipid imaging, and cell-type-resolved iron measurements could determine whether retinal endothelium, Müller glia, podocytes, tubular cells, macrophages, neurons, and Schwann cells respond similarly. Bulk tissue NRF2 or NLRP3 values may conceal opposing responses in neighboring cell types.

Third, disease stage and metabolic context are likely decisive. Early compensatory NRF2 activity may differ from late-stage exhaustion, and a compound that protects viable stressed cells may have limited effects after irreversible cell loss or fibrosis. Type 1 and type 2 diabetes, sex, age, kidney function, iron status, lipid profile, diet, and microbiota should be considered as biological modifiers rather than nuisance variables.

Fourth, dietary relevance must be improved. Many animal doses and in vitro concentrations cannot be achieved through normal food intake. Studies should distinguish habitual dietary exposure, fortified foods, standardized extracts, and pharmacological formulations. Comparative studies of parent compounds and circulating metabolites are essential, particularly for resveratrol and curcumin, whose native systemic bioavailability is low [108,109].

Fifth, NRF2 should not be treated as an invariably beneficial on-off switch. Chronic NRF2 activation may have cell-survival consequences that differ by tissue, and HO-1-associated iron release can become pro-ferroptotic when iron-buffering systems are insufficient [19]. Future studies should report NRF2 transcriptional activity, duration of activation, the relative induction of antioxidant versus iron-release, storage, and export genes, as well as the labile iron pool.

Finally, clinically meaningful combination strategies deserve to be studied. Polyphenols may be most useful when added to contemporary standard care and targeted to participants with high oxidative or inflammatory activity. Enrichment based on lipid-peroxidation signatures, inflammasome markers, metabolite exposure, or microbiome capacity could move the field toward precision nutrition. Adaptive and platform trials could compare chemically distinct polyphenol classes while sharing standardized mechanistic endpoints.

## 10. Conclusions

Taken together, ferroptosis and pyroptosis provide complementary explanations for how chronic metabolic stress becomes irreversible tissue injury in diabetic retinopathy, diabetic kidney disease, and diabetic peripheral neuropathy. NRF2 links these programs through regulation of glutathione, GPX4 support, lipid detoxification, iron handling, mitochondrial resilience, and redox-sensitive inflammatory signaling. Dietary polyphenols can engage several of these targets and therefore offer a plausible multi-pathway strategy.

Consistent with the preceding appraisal, the evidence is strongest in preclinical models. Corilagin, resveratrol, isoquercetin, quercetin, EGCG, punicalagin, and selected phenolic-rich extracts have shown direct or closely related effects on ferroptosis, pyroptosis, or NRF2 in diabetic tissues. Human studies provide encouraging but inconsistent signals for albuminuria, retinal edema, endothelial function, and neuropathic outcomes. They do not yet demonstrate that clinical benefit is mediated through the integrated ferroptosis–pyroptosis–NRF2 network.

Accordingly, the next phase should prioritize pathway specificity, metabolite-aware pharmacokinetics, cell and tissue resolution, standardized formulations, and clinically meaningful endpoints. Until such evidence is available, dietary polyphenols should be considered promising adjunctive modulators rather than validated substitutes for established microvascular prevention and treatment strategies.

## Figures and Tables

**Table 1:** Literature-identification framework used for this narrative integrative review.

**Search Domain**	**Representative PubMed Concepts**	**Evidence Prioritized**	**Principal Exclusions**
Organ-specific regulated cell death	(diabetic retinopathy OR diabetic kidney disease OR diabetic nephropathy OR diabetic peripheral neuropathy) AND (ferroptosis OR pyroptosis OR NLRP3 OR NRF2)	Primary cell and animal studies, human tissue data, mechanistic reviews	Studies without a diabetic complication model; isolated oncology findings without mechanistic relevance
Polyphenol–pathway interactions	(polyphenol OR flavonoid OR named compound) AND (ferroptosis OR pyroptosis OR NRF2 OR NLRP3) AND (diabetes OR hyperglycemia)	Chemically defined compounds or extracts with pathway readouts	Multicomponent formulas with no chemical characterization when a specific active constituent could not be inferred
Clinical translation	(polyphenol OR named compound OR polyphenol-rich extract) AND diabetic complication AND (trial OR randomized OR meta-analysis)	Controlled trials, prospective clinical studies, systematic reviews	Studies reporting only short-term glycemia without a complication-relevant endpoint
Foundational biology	Ferroptosis, gasdermin, inflammasome, KEAP1-NRF2, lipid peroxidation, iron metabolism	Seminal pathway-defining studies and authoritative reviews	Redundant mechanistic reports not needed to support the conceptual framework

**Table 2:** Distinguishing ferroptosis, pyroptosis, and NRF2-associated protection in diabetic tissues.

Dimension	Ferroptosis	Pyroptosis	NRF2-Associated Protection
Proximate event	Iron-dependent phospholipid peroxidation	Gasdermin pore formation and inflammatory lysis	Transcriptional adaptation to oxidative and electrophilic stress
Core machinery	SLC7A11, glutathione, GPX4, ACSL4, labile iron; complementary FSP1-CoQ, DHODH-CoQ, and GCH1-BH4 systems [30,32,34]	Sensors such as NLRP3, ASC, caspase-1 or caspase-4/5/11, GSDMD; caspase-3-GSDME in selected settings [11,42,43]	KEAP1-CUL3-NRF2-ARE; downstream GCLC/GCLM, NQO1, HMOX1, ferritin, ferroportin, and redox enzymes [16,17,19]
Stronger experimental evidence	Oxidized phospholipids or lipid ROS plus GPX4/SLC7A11/ACSL4 pattern and rescue by ferroptosis-selective inhibition	Cleaved gasdermin, membrane permeabilization, inflammatory caspase activity, and cytokine maturation	Nuclear NRF2 plus target-gene induction and loss-of-function or pathway-inhibition confirmation
Common but insufficient surrogate	Total ROS, MDA, iron, or PTGS2 alone	NLRP3, IL-1 beta, or caspase-1 expression alone	Total NRF2 protein without localization or transcriptional readout
Major diabetic triggers	Glutathione depletion, mitochondrial ROS, lipid overload, iron dyshomeostasis, TGF-beta, endoplasmic-reticulum stress	AGE-RAGE/NF-kappaB priming, TXNIP, mitochondrial danger signals, ATP-P2X7, ion flux, lipotoxicity	Oxidants and electrophiles that modify KEAP1; AMPK, SIRT1, PI3K-AKT, and kinase-dependent signaling
Important caveat	Multiple antioxidant systems can compensate for GPX4; total iron is not equivalent to labile iron	Inflammasome activation can occur without cell lysis; GSDME can bypass canonical NLRP3-GSDMD signaling	HO-1 can be protective or increase iron availability depending on context and buffering capacity

**Table 3:** Organ-specific evidence for the ferroptosis–pyroptosis–NRF2 network.

Complication	Vulnerable Compartments	Representative Ferroptosis Evidence	Representative Pyroptosis Evidence	Main Translational Limitation
Diabetic retinopathy	Retinal endothelium, pericytes, Müller cells, microglia, retinal pigment epithelium, neurons	Ferrostatin-1 rescue; ACSL4/autophagy regulation; microvascular dysfunction; NRF2-SLC7A11-GPX4 protection [49,50,52]	P2X7-NLRP3 activation and endothelial/glial inflammatory death [47,54]	Human trials measure edema, visual function, and systemic oxidation rather than lipid- or gasdermin-specific markers
Diabetic kidney disease	Podocytes, glomerular endothelium, mesangial cells, tubular epithelium, macrophages	Human biopsy xCT/GPX4 reduction; lipid peroxidation and ferrostatin-1 rescue; NRF2-dependent protection [40,70,71]	Caspase-11/4-GSDMD podocyte injury and NLRP3-associated renal inflammation [72,73]	Kidney disease stage, background therapy, and cell-specific effects are heterogeneous; hard renal outcomes are scarce
Diabetic peripheral neuropathy	Schwann cells, sensory neurons, axons, endoneurial vessels, macrophages	High-glucose-induced Schwann-cell ferroptosis linked to NRF2 suppression; honokiol rescue [94,95]	ROS-NLRP3-associated Schwann-cell pyroptosis and neuroimmune signaling [48,96]	Very limited human tissue validation; pain scores do not distinguish axonal repair from symptomatic change
Diabetic wound/vascular repair	Endothelium, macrophages, fibroblasts, keratinocytes	Iron and lipid-peroxidation changes with NRF2-associated response to ferroptosis inhibition [104]	Inflammatory cell death may impair repair, but direct dual-pathway studies remain limited	Supportive model rather than a canonical microvascular endpoint

**Table 4:** Representative dietary polyphenols and phenolic compounds relevant to the proposed network.

Compound/Class	Representative Dietary Source	Principal Proposed Targets	Diabetic Complication Evidence	Evidence Level and Caveat
Resveratrol (stilbene)	Grapes, berries, peanuts, wine	SIRT1, AMPK, NRF2, HMGB1, mitochondrial ROS, NF-kappaB	Ferroptosis-related retinal protection; NRF2-associated renal protection; experimental neuropathy [56,85,97]	Direct preclinical pathway evidence plus small human renal trials; unconjugated bioavailability is very low
Curcumin (diarylheptanoid)	Turmeric	KEAP1-NRF2, NF-kappaB, NLRP3, mitochondrial and fibrotic signaling	Retinal oxidative protection; albuminuria and NRF2 response in DKD; mixed DPN trials [60,89,102]	Formulation-dependent; direct regulated-cell-death evidence is weaker than general redox evidence
Quercetin/isoquercetin (flavonols)	Onions, apples, berries, tea	NRF2-HO-1, p53-SLC7A11-GPX4, NLRP3, autophagy	Anti-ferroptotic kidney and retinal effects [57,58,74]	Strong preclinical evidence; no definitive microvascular outcome trial with pathway biomarkers
EGCG/green tea catechins	Green tea	KEAP1-NRF2, glutathione defense, podocyte survival, inflammatory signaling	Experimental DKD protection and reduced residual albuminuria [76,87]	Human albuminuria signal; pathway-specific human evidence absent
Corilagin/punicalagin (hydrolyzable tannins)	Pomegranate and tannin-rich plant foods; medicinal plant extracts	NRF2-GPX4 and TXNIP-NLRP3-GSDMD	Direct anti-ferroptotic DR and anti-pyroptotic DKD evidence [55,80]	Compelling mechanistic animal/cell data; dietary exposure and clinical pharmacokinetics uncertain
Anthocyanins/proanthocyanidins	Berries, grapes, pigmented fruits, pine bark extract	Endoplasmic-reticulum stress, microglia, endothelial barrier, redox signaling	Retinal protection and preliminary human DR benefits [62,65,69]	Extract composition varies; clinical studies lack ferroptosis/pyroptosis biomarkers
Naringenin, vitexin, baicalin	Citrus and selected plant foods or extracts	SIRT1-FOXO3a, GPX4, NRF2-MAPK	Retinal neuroprotection and renal ferroptosis/redox modulation [64,77,79]	Predominantly preclinical; doses often exceed habitual dietary exposure
Honokiol and carnosic acid	Magnolia-derived preparations; rosemary/sage	SIRT3, mitochondrial ROS, SIRT1, ferroptosis/pyroptosis-related signaling	DKD pyroptosis and DR/DPN neurovascular protection [61,82,95]	Phenolic candidates with preclinical evidence; not established dietary therapies

Note: Representative dietary sources indicate chemical origin, not exposure equivalence. Most mechanistic studies used purified compounds or concentrated extracts, often at doses or concentrations exceeding those achievable through habitual food intake; foods, standardized extracts, conventional supplements, and formulation-enhanced products should therefore not be treated as interchangeable.

**Table 5:** Selected human studies on polyphenol-rich interventions in diabetic microvascular complications.

Complication and Study Design	Intervention	Duration/Sample	Main Reported Outcome	Interpretation for the Proposed Network
Early DR, placebo-controlled study [65]	French maritime pine bark extract (Pycnogenol)	3 months; 46 participants	Improved retinal edema, retinal arterial flow, and visual acuity versus placebo	Supports vascular and antioxidant benefit; no pathway-specific biomarkers
Vascular retinopathy, randomized double-blind phase plus open phase [66]	Pycnogenol 50 mg three times daily	2 months; 40 participants overall	Reduced leakage and deterioration; improved vascularization and visual measures	Small, mixed retinal diagnoses and older methods limit certainty
Non-proliferative DR, randomized study [67]	Pycnogenol 50 mg + vitamin E 30 mg + coenzyme Q10 20 mg	6 months; 68 participants	Reduced circulating ROS and central macular thickness	Multicomponent intervention prevents attribution to polyphenols
Chronic diabetic macular edema, open-label pilot [68]	Curcumin-phospholipid formulation twice daily	At least 3 months; 11 participants/12 eyes	Improved visual acuity and reduced OCT edema in most eyes	Very small uncontrolled pilot; formulation exposure not linked to NRF2/ferroptosis
Mild-moderate non-proliferative DR, randomized placebo-controlled trial [69]	Maltodextrinated grape pomace extract formulation	6 months; 99 participants	Improved best-corrected visual acuity and retinal thickness; reduced dROMs and oxidized LDL	Promising contemporary clinical signal; compound-specific and regulated-cell-death effects unresolved
DKD with residual albuminuria, double-blind randomized trial [87]	Green tea polyphenols containing 800 mg EGCG	12 weeks; 42 participants	41% UACR reduction versus 2% increase with placebo	Supports podocyte/renal effect; ferroptosis and pyroptosis were not measured in participants
Overt type 2 diabetic nephropathy, randomized double-blind trial [88]	Turmeric 500 mg three times daily (22.1 mg curcumin per capsule)	2 months; 40 participants	Reduced proteinuria, TGF-beta, and IL-8	Relevant anti-inflammatory signal; small and short-term
Type 2 diabetes with renal albumin excretion, self-controlled intervention [89]	Curcumin 500 mg/day	15–30 days	Reduced urinary microalbumin and MDA; increased lymphocyte NRF2/NQO1	Direct human NRF2 signal, but no placebo group and very short exposure
Type 2 diabetes with albuminuria, randomized double-blind trial [90]	Resveratrol 500 mg/day added to losartan	90 days; 60 participants	Reduced UACR; no significant eGFR or creatinine change	Clinical renal signal without regulated-cell-death measurements
Stage 3 CKD and diabetes, randomized crossover trial [91]	Resveratrol 400 mg/day	6 weeks; 28 participants	Improved brachial flow-mediated dilation; no eGFR or HbA1c change	Demonstrates vascular target engagement, not renal ferroptosis/pyroptosis
Type 2 diabetes with DPN, randomized double-blind trial [100]	Nanocurcumin 80 mg/day	8 weeks; 80 participants	Reduced neuropathy severity and selected glycemic measures	Favorable but needs replication with objective nerve and exposure measures
Type 2 diabetes with DPN, randomized double-blind trial [102]	Nanocurcumin 40 mg twice daily	16 weeks; 97 randomized	No benefit for pain, MNSI examination, NDS, or metabolic outcomes	Important negative replication; argues against assuming class-wide efficacy
Diabetic peripheral/autonomic neuropathy, double-blind controlled trial [103]	Cocoa capsules providing 50 mg polyphenols	12 weeks; 39 participants	No significant superiority for clinical or somatosensory outcomes	Dose, power, and phenotype may have limited effect detection

**Table 6:** Proposed translational framework for future polyphenol trials.

Layer	Recommended Measures	Purpose
Intervention identity	Full phytochemical profile, batch testing, stability, food matrix, formulation, dose, and adherence	Distinguish compound, extract, and delivery-system effects
Exposure	Parent compound, conjugates, microbial metabolites, and time-resolved pharmacokinetics	Confirm that participants and target tissues are plausibly exposed
Ferroptosis	Oxidized phospholipids, 4-hydroxynonenal adducts, GPX4 activity, SLC7A11, ACSL4, labile-iron or iron-handling panel	Move beyond nonspecific MDA/ROS measurements
Pyroptosis	Cleaved GSDMD/GSDME, caspase-1 or caspase-4/5 activity, ASC specks, IL-1 beta, IL-18, membrane-injury markers	Distinguish inflammasome activation from executed pyroptosis
NRF2 target engagement	Nuclear NRF2, NQO1, GCLC/GCLM, HO-1 plus ferritin and ferroportin	Verify transcriptional activity and detect context-dependent iron effects
Organ outcome	OCT/perfusion/vision; UACR/eGFR/tubular injury; nerve conduction/small-fiber structure/pain and function	Establish clinically meaningful benefit beyond biomarker movement
Design safeguards	Adequate power, preregistration, placebo and standard-care control, sex and diabetes-type stratification, sufficient duration	Reduce bias and improve generalizability

## Data Availability

No new data were created or analyzed in this study. Data sharing is not applicable to this article.

## Abbreviations

ACSL4	acyl-CoA synthetase long-chain family member 4
AGE	advanced glycation end product
AMPK	AMP-activated protein kinase
ARE	antioxidant response element
ASC	apoptosis-associated speck-like protein containing a CARD
BCVA	best-corrected visual acuity
CKD	chronic kidney disease
DKD	diabetic kidney disease
DPN	diabetic peripheral neuropathy
DR	diabetic retinopathy
EGCG	epigallocatechin gallate
eGFR	estimated glomerular filtration rate
FSP1	ferroptosis suppressor protein 1
GPX4	glutathione peroxidase 4
GSDMD/GSDME	gasdermin D/gasdermin E
HO-1	heme oxygenase 1
KEAP1	Kelch-like ECH-associated protein 1
MDA	malondialdehyde
NF-kappaB	nuclear factor kappa B
NLRP3	NLR family pyrin domain containing 3
NQO1	NAD(P)H quinone dehydrogenase 1
NRF2	nuclear factor erythroid 2-related factor 2
OCT	optical coherence tomography
ROS	reactive oxygen species
SIRT1	sirtuin 1
SLC7A11	solute carrier family 7 member 11
TXNIP	thioredoxin-interacting protein
UACR	urinary albumin-to-creatinine ratio
